# Analysis of the sol and gel structures of potato starch over a wide spatial scale

**DOI:** 10.1002/fsn3.2441

**Published:** 2021-07-21

**Authors:** Akane Nagasaki, Go Matsuba, Yuka Ikemoto, Taro Moriwaki, Noboru Ohta, Keiichi Osaka

**Affiliations:** ^1^ Graduate School of Organic Materials Engineering Yamagata University Yonezawa Japan; ^2^ SPring‐8/JASRI Sayo‐gun Japan

**Keywords:** amylopectin, FT‐IR, potato starch, X‐ray scattering

## Abstract

We analyzed edible potato starch and observed the interaction between its granular structure and water molecules. We studied the changes caused by gelatinization during heating and stirring using microscopy, micro‐FT‐IR spectroscopy, and X‐ray scattering techniques. A wide range of spatial scales was revealed using these various techniques. The rate of gelatinization varied significantly and was dependent on the starch concentration. The process of adsorption of water on starch molecules was studied using the humidity‐controlled FT‐IR spectroscopy technique. Furthermore, by comparing the X‐ray scattering profiles of dry and wet granules, the 9‐nm repeat “cluster” structure was studied. A gradual collapse of the granules occurred during the processes of heating and stirring. A clustered smectic structure and a smectic‐like structure were observed in the opaque gel after gelatinization. Upon further heating, a transparent gel was obtained after the melting of the cluster.

## Graphical abstract

We analyzed edible potato starch and observed the interaction between its granular structure and water molecules. A wide range of spatial scales was revealed using various techniques such as X‐ray scattering and FT‐IR measurements. We could succeed gelatinization and gelation process with a wide spatial scale.

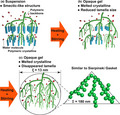

## 1 INTRODUCTION

Starch is composed of amylose (an essentially linear or scarcely branched glucan) and amylopectin (a highly branched glucan), with both components accounting for 15%–30% and 70%–85% of starch, respectively (Jane et al., 1997; Kerr, 1950; Nakamura et al., 2020; Sterling, 1968). The fine structure of amylopectin differs significantly from the structure of glycogen. Amylopectin has a unit structure designated as a cluster, whereas glycogen exhibits no such structure because the α‐1,6 branch points in glycogen are randomly distributed. Amylopectin clusters are interconnected by long chains that span across two or three clusters. The short and intermediate chains within a single cluster are called nonbranched chains or branched chains (Jane et al., 1997; Peat et al., 1956).

Starch is a vital source of energy that helps sustain human life. Starch that stores nutritive energy in plant roots, tubers, or endosperms such as potato, tapioca, corn, and rice primarily comprises of carbohydrates in the form of polysaccharides. On the micrometer scale, starch granules ∼1–100 µm in size have a growth‐ring structure composed of alternately arranged semicrystalline and amorphous shells. The semicrystalline shells are composed primarily of branched‐chain amylopectin clusters, in contrast to the amorphous shells, which contain long linear‐chain amylose and low‐molecular‐mass amylopectin. On the nanometer scale, each semicrystalline shell consists of lamellae of alternating crystalline and amorphous layers, with a typical lamellar spacing of ~9 nm (also known as the 9‐nm repeat structure), with crystallinity ranging from 15% to 45%. At the atomic level, the packing of the crystal structure of starch can be classified as either monoclinic (A‐type) or hexagonal (B‐type) packing of double helices of amylopectin or amylose chains (Gidley & Bulpin, 1987; Kainuma & French, 1972).

In this study, we analyzed edible potato starch, which is commonly used by humans. Swollen granules with exceptionally large volumes in native potato starch exhibit high viscosity that can be attributed to their high swelling capacities. There is a considerable interest in employing physical and enzymatic methods to improve starch properties to address food safety and environmental issues that can be attributed to the use of chemicals for starch modification (Wang et al., 2018). Colussi et al. (2020) investigated the effects of heat–moisture treatment on the hydrostatic pressure and evaluated their effects on various characteristics and in vitro digestibility of potato starch. When starch granules are heated in the presence of excess water, they swell and rupture when the semicrystalline structure melts during gelation. This results in increased solution viscosity. During the subsequent cooling process, viscosity further increases during the gelation of the aqueous amylopectin–amylose mixture.

In particular, gelatinization is quite significant for controlling the stiffness and transparency of edible starch gels, such as warabi‐mochi and other Japanese sweets. It is necessary to quantitatively analyze their properties and the changes in the properties (attributable to the melting of the crystalline structure), to better understand the process of starch gelatinization.

In this study, the evolution of the nanocrystalline structure of native potato starch granules was revealed using the simultaneous small/wide‐angle X‐ray scattering (SAXS/WAXS), ultra‐small‐angle X‐ray scattering (USAXS), and Fourier transform‐infrared (FT‐IR) spectroscopy techniques. The results were quantitatively correlated with the corresponding changes in the characteristics.

## 2 EXPERIMENTAL

### 2.1 Starch samples

Native starch powder (Kona‐fubuki, Awano Agri‐Foods, Sendai, Japan) samples were purchased from a local supermarket. Distilled water was used to prepare the samples. The powder samples were used at various humidity levels. The dry powder and hydrated slurries under excess water were placed in a 0.5‐mm‐thick sample cell sandwiched between Kapton^®^ films for X‐ray scattering measurements. First, to quantitatively evaluate the process of gelatinization, potato starch dispersions of various concentrations were prepared, and the changes in shape, hardness, and transparency with temperature and time were recorded using various techniques.

**FIGURE 1:**
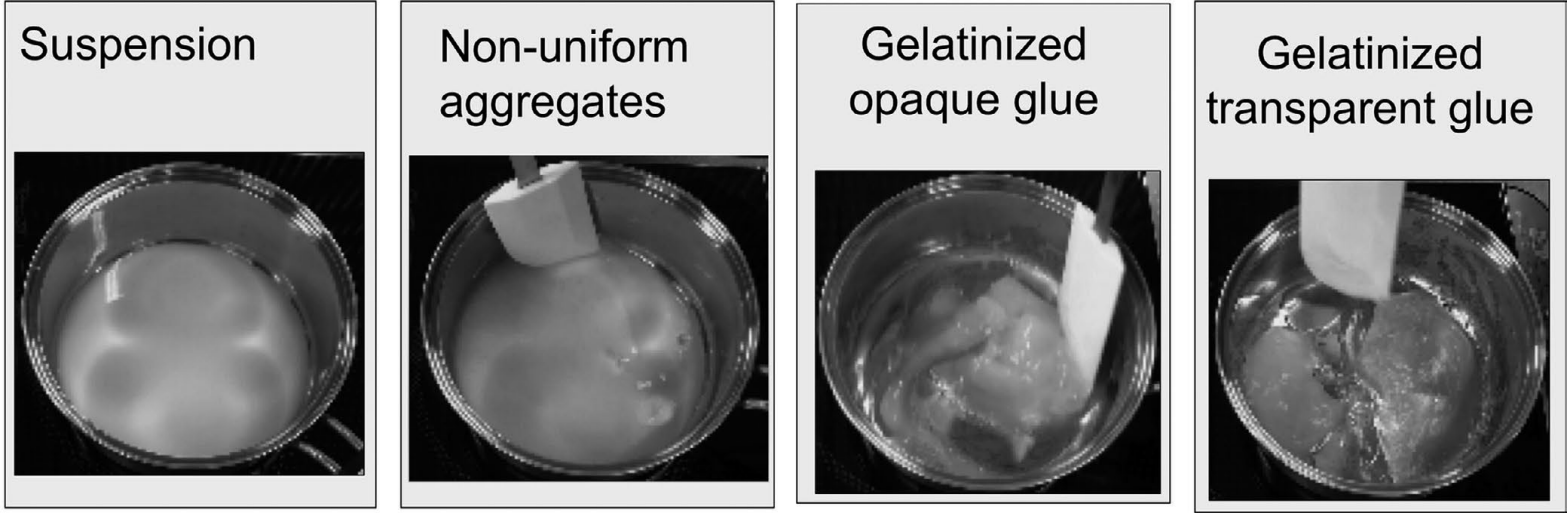
Images showing the time evolution of starch gelatinization (from suspension to transparent glue; heating at 60°C) when the sample was manually stirred using a plastic spatula

Figure 1 shows gelatinization of a starch dispersion upon heating at 60°C using an induction cooker (KZ‐PH33, Panasonic Co. Ltd., Osaka). The sample was manually stirred using a plastic spatula. A video camera was used to quantitatively evaluate the extent of gelatinization by recording the changes in the visual appearance (with heating and stirring times) of the suspension. Owing to uniform gelanitization, the suspension was manually stirred using a plastic spatula. When the suspension was being heated and stirred, “nonuniform aggregates” were formed, which subsequently turned into an opaque glue (sol state). Eventually, a transparent glue was formed (sol state).

### 2.2 Apparatus

The optical microscopy technique was used to study the samples using a Keyence VW‐5000 microscope (Osaka, Japan) equipped with a high‐speed CCD camera (Zhao et al., 2012, 2013). A Linkam CSS‐450 high‐temperature cell (Surrey, UK) was used to control the temperature.

SAXS and WAXS measurements were performed on a Nano‐viewer system (Rigaku Co., Tokyo, Japan) at a wavelength of 0.154 nm (CuK*α*). The camera lengths were 750 and 110 mm for SAXS and WAXS, respectively. A Pilatus 1 M (Dectris AG, Baden, Switzerland) detector was used, with a *q* range of 0.15 to 1.5 nm^‐1^ for SAXS and 3.5 to 25 nm^‐1^ for WAXS; *q* is the magnitude of the scattering vector and is defined as follows:

(1) $$q=\frac{4\pi }{\lambda }\sin\theta ,$$

where 2θ and λ are the scattering angle and wavelength, respectively. The sample thickness was ~500 μm. Data processing, which included controlling the contrast of the 2D patterns and the preparation of a 1D profile from the obtained 2D patterns, was performed using the FIT‐2D software (Ver. 12.077, Andy Hammersley/ESRF, Grenoble, France).

The time‐resolved SAXS and WAXS measurements were performed using the BL40B2 beamline (SPring‐8, Hyogo, Japan) at a wavelength of 0.10 nm (Masunaga et al., 2007). The camera lengths were 2,300 and 130 mm for SAXS and WAXS, respectively. A Pilatus3 S 2M detector with a *q* range of 0.07 to 3.0 nm^‐1^ for SAXS and a flat panel detector (C9728DK‐10, Hamamatsu Photonics K.K., Hamamatsu, Japan) with a *q* range of 3.5 to 23 nm^‐1^ for WAXS were used.

USAXS measurements were performed using the BL19B2 beamline (developed at SPring‐8; Osaka et al., 2016) at an incident X‐ray beam wavelength of 0.068 nm. The camera lengths for USAXS were set to 40.77 m. The 2D USAXS profiles were obtained using a Pilatus‐2M two‐dimensional detector (Dectris Ltd., Baden, Switzerland). The scattering vector, *q*, was recorded between 3 × 10^−3^ and 0.6 nm^−1^.

Microbeam FT‐IR measurements were performed using the BL43IR beamline (SPring‐8, Hyogo, Japan; Ikemoto et al., 2012). A Bruker FT‐IR 120HR/X spectrometer with a wide spectral range (100–20,000 cm^‐1^) was used. The maximum wavenumber resolution was 0.0063 cm^‐1^. An in‐house humidity control cell connected to a humidity generator (HUM‐1, Rigaku, Tokyo) was installed in the IR microscope of the BL43IR beamline. A 1‐mm‐thick barium fluoride was used as the IR window (Ikemoto et al., 2004). The images of the microbeam FT‐IR spectrometer with the humidity control cell and the granular starch samples are shown in Figure S1 (Supporting Information).

## 3 RESULTS

### 3.1 Gelatinization process

**FIGURE 2:**
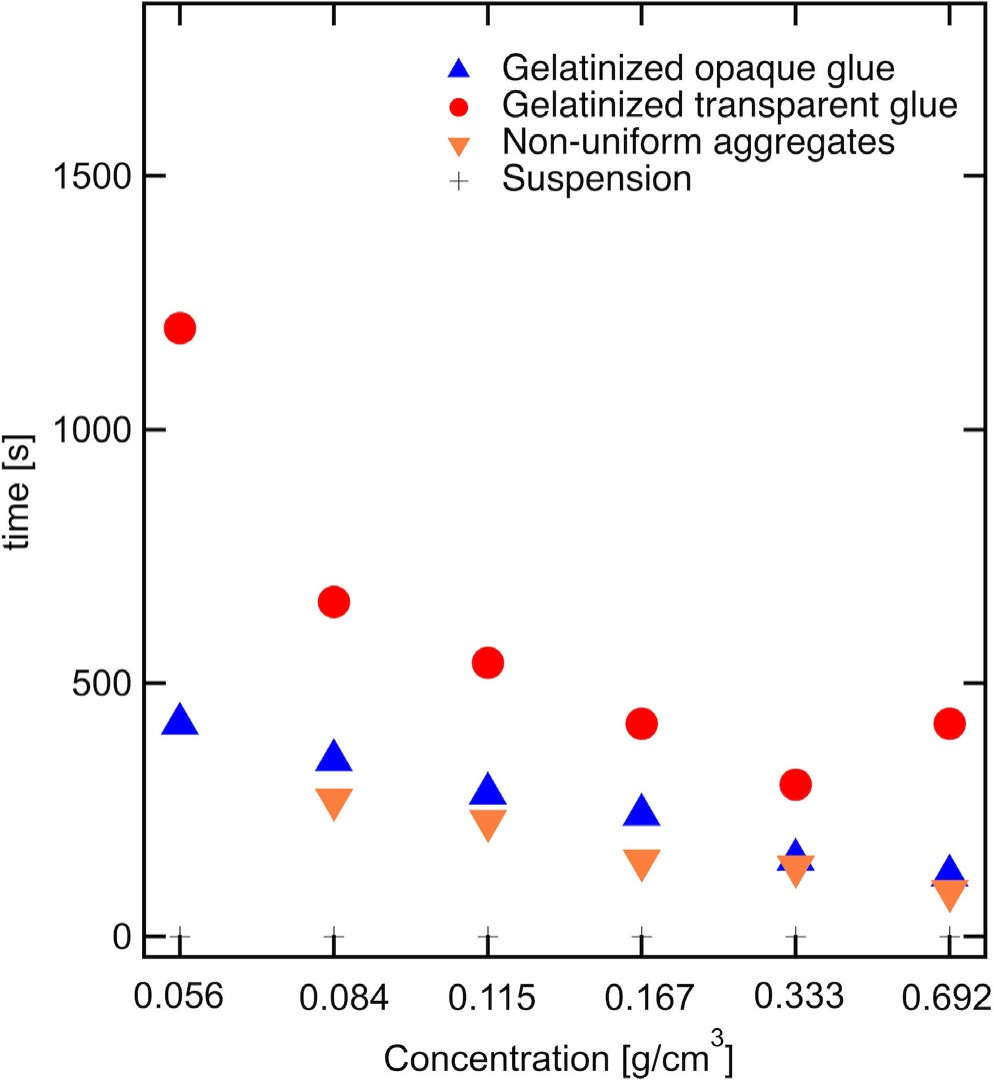
Dependence of concentration of starch suspensions on heating and stirring times, featuring nonuniform aggregates and transparent and opaque glue forms

Figure 2 shows the dependence of suspension concentration on the heating time. The formation time of the nonuniform aggregates, opaque glue, and transparent glue is observed to decrease with increasing starch concentration. Amylopectin molecules in the swollen starch granules absorb water and form networks during the subsequent suspension heating process. We could observe the formation of the nonuniform aggregates when the sample was heated for some time (Figure 1). The amylopectin molecular networks are assembled locally and macroscaled inhomogeneously. At low concentrations (below 0.07 g/cm^3^), nonuniform aggregates are not observed, and gelatinization occurs directly from the suspension state. In this case, expanded amylopectin chains might have difficulty in overlapping with each other. When the networks overlap, gelatinization occurs. This network structure can spread throughout the entire system. The opaque glue (sol state) could be observed. Further heating resulted in the formation of transparent glue, that is, the micron‐scaled density fluctuations disappeared. At low starch concentrations, slow gelatinization ensues, which can be attributed to the slow diffusion of the amylopectin molecules. Therefore, gelatinization significantly helps in the diffusion of the amylopectin molecular chains and network formation. Furthermore, the heating and stirring times of the transparent glue (the other sol state) increased at lower starch concentrations. The transparent glue is formed by diffusion. For a more detailed analysis of gelatinization from starch granules, their structural formation from water swelling is analyzed using the wide‐scaled structural analysis method.

**FIGURE 3:**
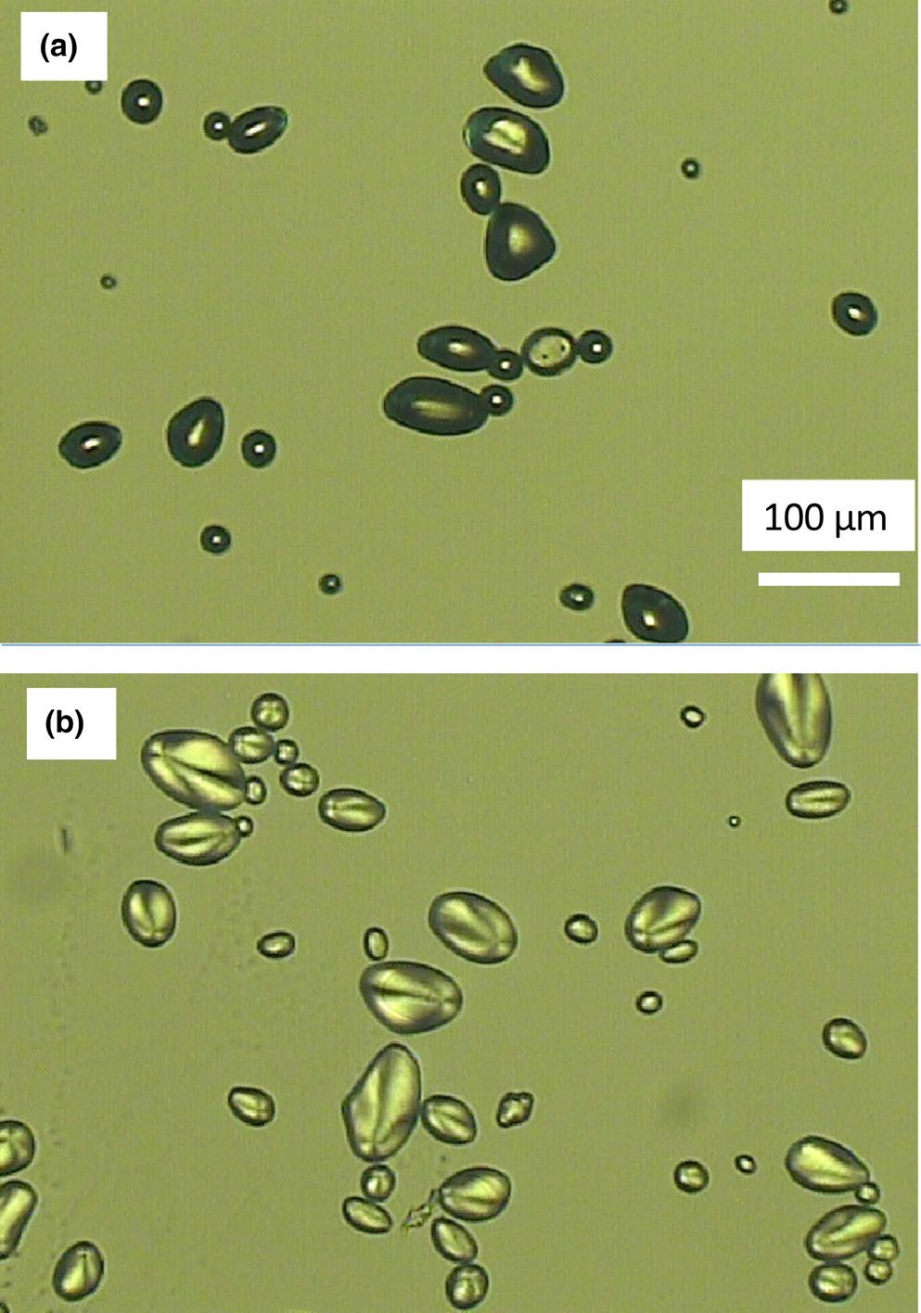
Optical microscopy images of starch granules (a) before and (b) after swelling

Figure 3a,b shows the optical microscopy images of dry and swelled starch samples, respectively. The granular size is ~20–70 µm in the dry state and ~35–100 µm after swelling. The water absorption capacity of potato starch is ~75%, according to a previous report (Yadav et al., 2016).

**FIGURE 4:**
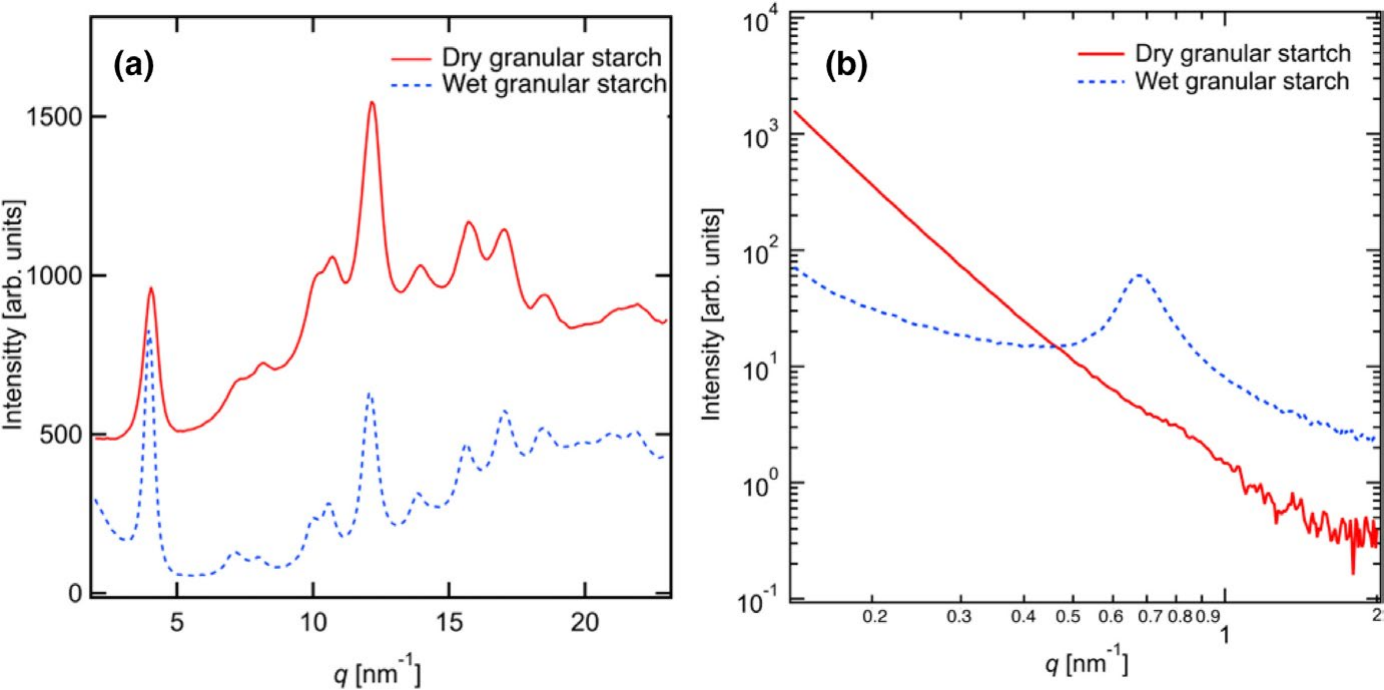
Scattering profiles of dry and wet potato starch samples obtained using the (a) WAXS and (b) SAXS techniques

Furthermore, WAXS and SAXS measurements were performed for the dry and wet granular starch samples. In Figure 4a, the crystal diffraction peaks in each granular starch sample can be observed with WAXS peaks at *q* values of 4.0, 7.9, 10.7, 12.1, 14.0, 15.8, 17.0, and 18.6 nm^‐1^; these values agree with those reported in a previous study (Gunaratne & Hoover, 2002), thus validating the B‐type crystalline structure of this starch sample. After swelling, the intensity of the reflection peak of (001), around *q* = 4.0 nm^‐1^, in the wet sample increased; however, the reflection peak of (132) decreased (Sarko & Wu, 1978; Singh et al., 2007; Takahashi et al., 2004). This suggests that the amylopectin helix is aligned in the chain direction owing to swelling, whereas the *a*‐axis and *b*‐axis direction of the amylopectin crystal become disordered.

Figure 4b shows the SAXS profiles of the dry and wet granular starch samples. The correlation length is evaluated using the peak position, *q*_peak_, as follows:

(2) $$L=\frac{2\pi }{q_{\mathrm{peak}}}.$$

In the dry starch sample, a shoulder‐type peak (*q*_peak_; ~0.81 nm^‐1^) is observed, with a corresponding correlation length of 8.27 nm (the 9‐nm repeat structure that is a cluster in the amylopectin molecules). After swelling, the peak shifted to the low‐*q* region (*q* = 0.67 nm^‐1^, *L* = 9.38 nm) in the wet starch sample. The peak became clearer and the peak intensity increased. This suggested that the water molecules could be absorbed into the amorphous region between the crystal lamellae. Furthermore, we attempted to evaluate the shape of the amylopectin molecules (at the nanometer scale) from the slope. The scattering intensity decreases with increasing *q*, in accordance with the power‐law formula expressed below in Equation (3):

(3) $$I\left(q\right)∼I^{‐k}.$$

When 3 ≤ *k* ≤ 4, the scattering exponent, *k*, is related to the surface fractal dimension, *d*_s_, through the following equation

(4) $$k=6‐d_{s}$$

in a *d*_s_‐dimensional space. In a 3D space, *d*_s_ ranges from 2 to 3, which corresponds to *k* ranging from 4 to 3. In the case of a smooth surface, the surface fractal dimension is defined by *d*_s_ = 2; therefore, *k* = 4. This is known as Porod's law. The profile of the dry potato starch sample in the low‐*q* region is described by *I*(*q*) ~ *q* ^−3.9^. The scattering profile originates from the smooth interface as *d*_s_ = 2.1. This interface is believed to be derived from micron‐scale aggregates of amylopectin, such as those in dry granular samples.

**FIGURE 5:**
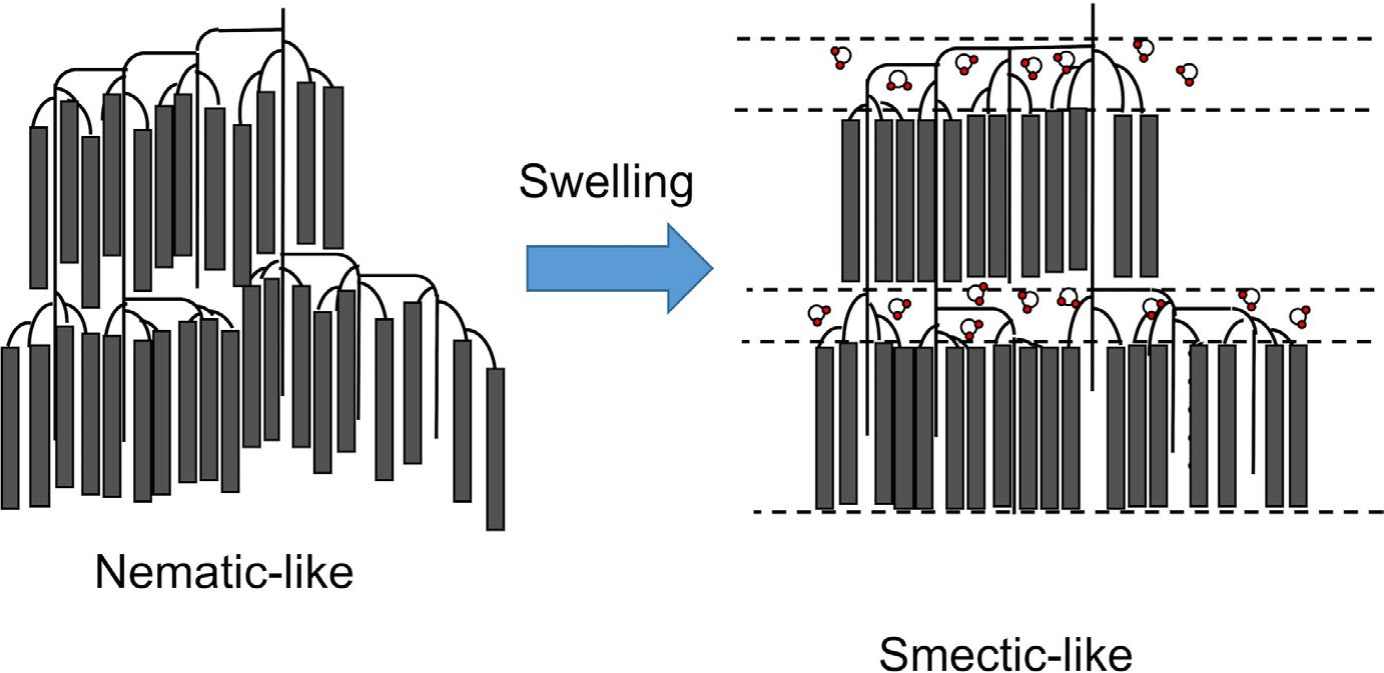
Schematic representation of the 9‐nm repeat structure. The gray rectangle represents the double helix structure composed of glucose. Before swelling, the amorphous regions between crystal lamellae get shrunken, and the higher‐order structure becomes “nematic‐like.” After swelling, water molecules expand into the interlamellar region, and the resulting higher‐order structure is “smectic‐like”

Analysis of the results obtained using the SAXS technique revealed that the granular starch samples expanded by ~114% from the 9‐nm repeat structure. Moisture content obtained using a heat drying type moisture meter (ML‐50, A&D Co. Ltd.) was found to be ~50% after 24 hr of absorption at 25°C. This is consistent with the moisture content evaluated based on the changes in the long period length in rice starch (Jane et al., 1997). Therefore, before swelling, the amylopectin helix is aligned in the chain direction, similar to a disordered nematic‐like structure (Blazek & Gilbert, 2011; Daniels & Donald, 2003, 2004). On the other hand, the correlation between the amorphous and crystal regions is observed after swelling. This can be attributed to the diffusion of water molecules into the amorphous part between the crystalline layers of the amylopectin helix. This results in a similar appearance of the higher‐order structure and smectic‐like phases. Figure 5 shows the schematic model of amylopectin during swelling.

For a more detailed insight into the swelling of starch powders, the in situ FT‐IR spectroscopy technique (with humidity control) was used for sample analysis.

### 3.2 FT‐IR measurements in the humidity control cell

**FIGURE 6:**
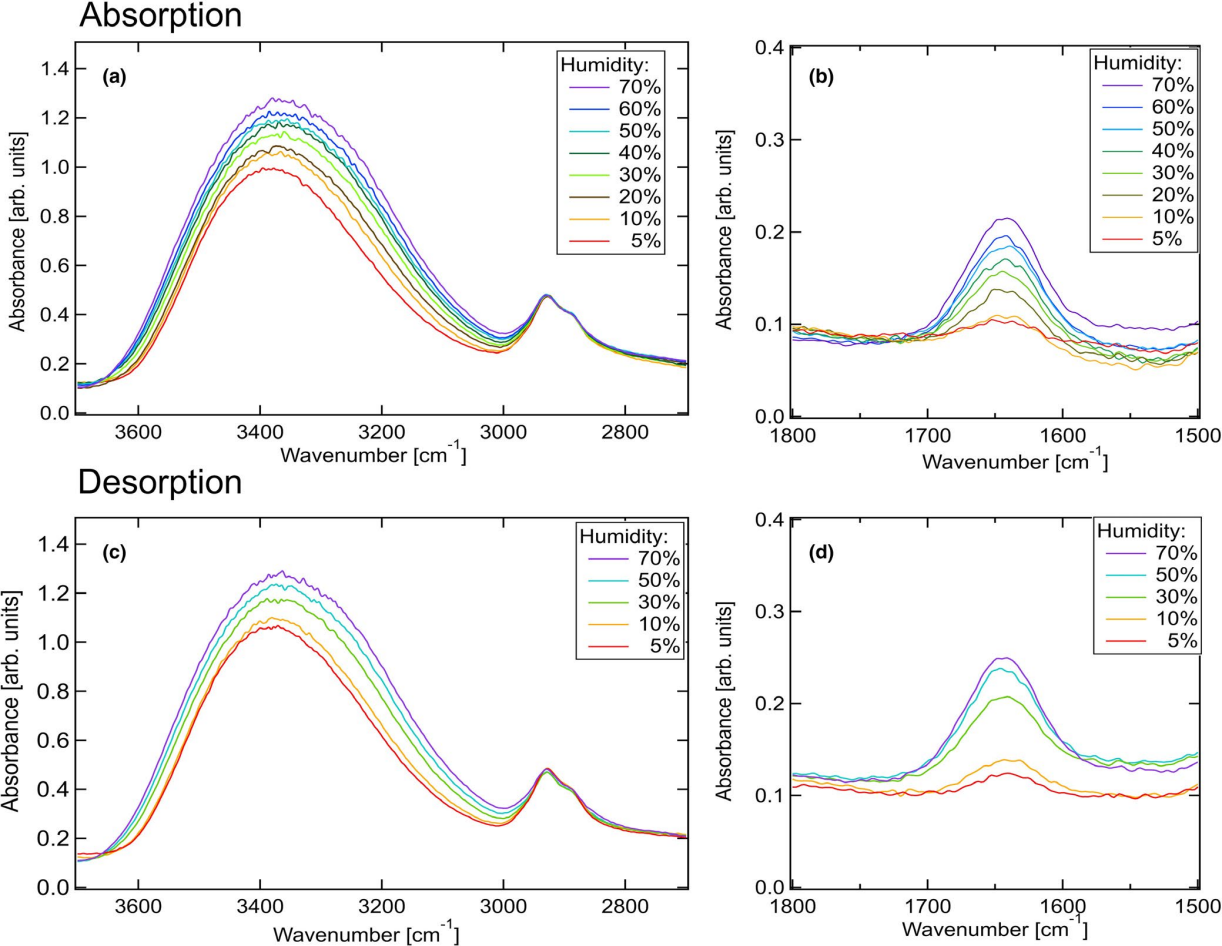
FT‐IR spectral profiles (absorption) in the regions spanning from (a) 3,700 to 2,700 cm^‐1^ and (b) 1,800 to 1,500 cm^‐1^. FT‐IR spectral profiles recorded during desorption in the regions spanning from (c) 3,700 to 2,700 cm^‐1^ and (d) 1,800 to 1,500 cm^‐1^

Figure 6a,b shows the FT‐IR spectral profile in the equilibrium condition under varying humidity conditions. The peak at approximately 3,400 cm^‐1^ (Figure 6a) corresponds to the OH stretch of starch and water molecules (Flores‐Morales et al., 2012). Furthermore, two absorption bands appear at 2,926 and 2,856 cm^‐1^, which are assigned to the vibrations of CH_2_. In Figure 6b, the band at 1,640 cm^‐1^ is assigned as a feature of tightly bound water present in the starch (Fang et al., 2002). The peak intensity at 3,400 cm^‐1^ (Figure 6a) increases and shifts to a comparatively low wavenumber region with increasing humidity. In Figure 6b, the peak intensity at 1,640 cm^‐1^ increases gradually with increasing humidity. These results suggest that starch, specifically the amylopectin molecules, absorbed the water molecules exploiting the strong hydrogen bonding interactions between amylopectin and water molecules. On the other hand, the bands at approximately 2,900 cm^‐1^ (Figure 6a) are almost independent of humidity. The results suggest that the CH_2_ bonds present in the main chain are not significantly affected by hydrogen bonds.

Figure 6c,d shows the FT‐IR spectral profiles during desorption. The band intensities at 3,360 cm^‐1^ in Figure 6c decrease with decreasing humidity and shift to the high wavenumber region. The band intensities at 1,640 cm^‐1^ in Figure 6d also decrease. The extent of hydrogen bonding between amylopectin and water is reduced during desorption. Furthermore, the bands at 2,900 cm^‐1^ are independent of humidity.

**FIGURE 7:**
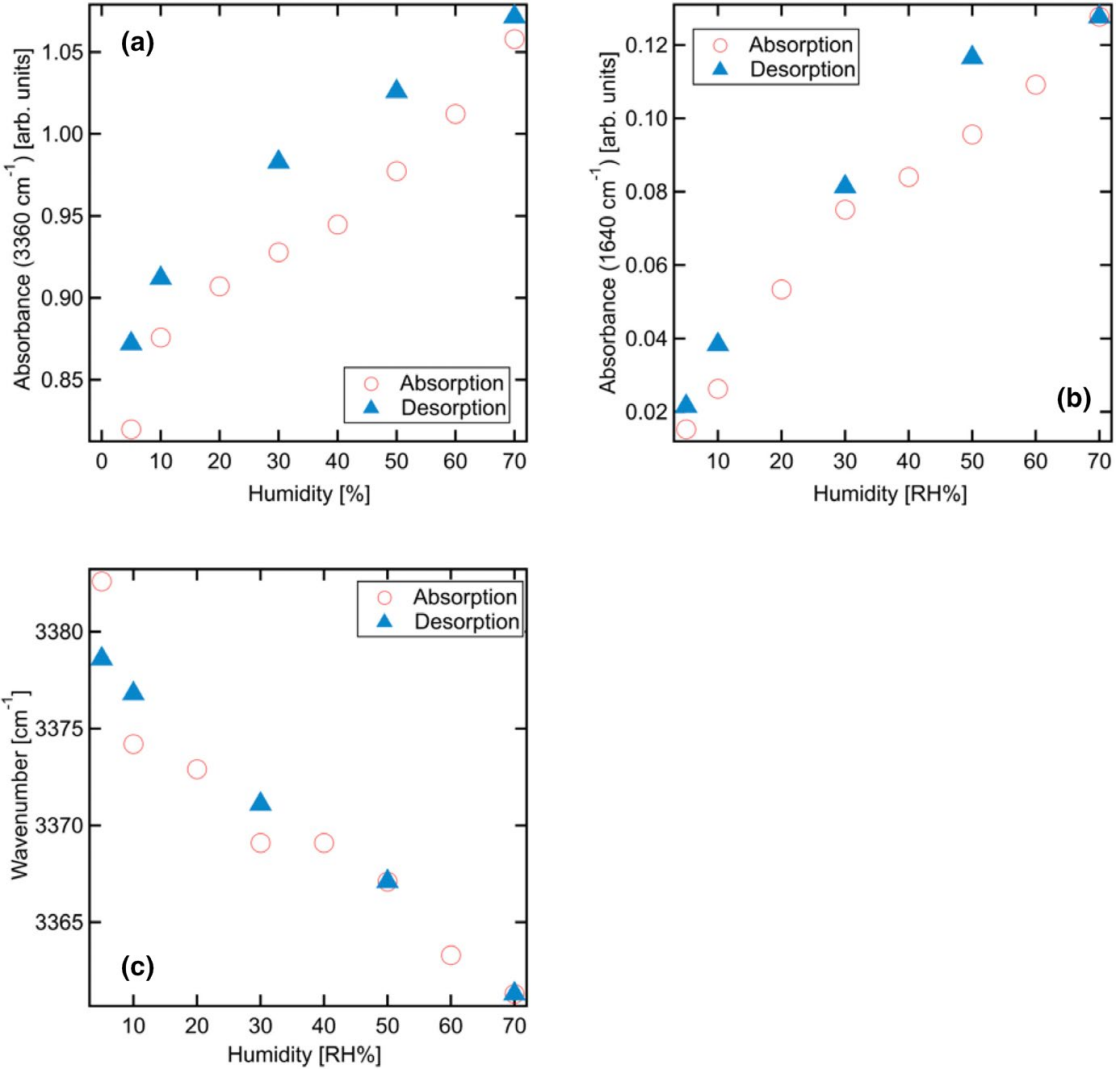
Humidity dependence of (a) the absorbance at approximately 3,360 cm^‐1^, (b) the absorbance at approximately 1,640 cm^‐1^, and (c) the wavenumber at approximately 3,400 cm^‐1^

Figure 7a,b has been analyzed to discuss the humidity dependence of these bands. Figure 7a,b shows the values of the intensity of absorbance for both absorption and desorption under various humidity conditions. Peak absorbance values increase almost linearly with humidity; however, they are consistently higher during desorption than those during absorption. This can be attributed to the relatively slow desorption process, which involves the removal of water molecules from amylopectin. Wavenumbers corresponding to the peak absorbance values at 3,360 cm^‐1^ are correlated with humidity in Figure 7c. These wavenumbers, for both the absorption and desorption processes, almost coincide.

### 3.3 Structures of potato starch suspensions, aggregations, and gels

**FIGURE 8:**
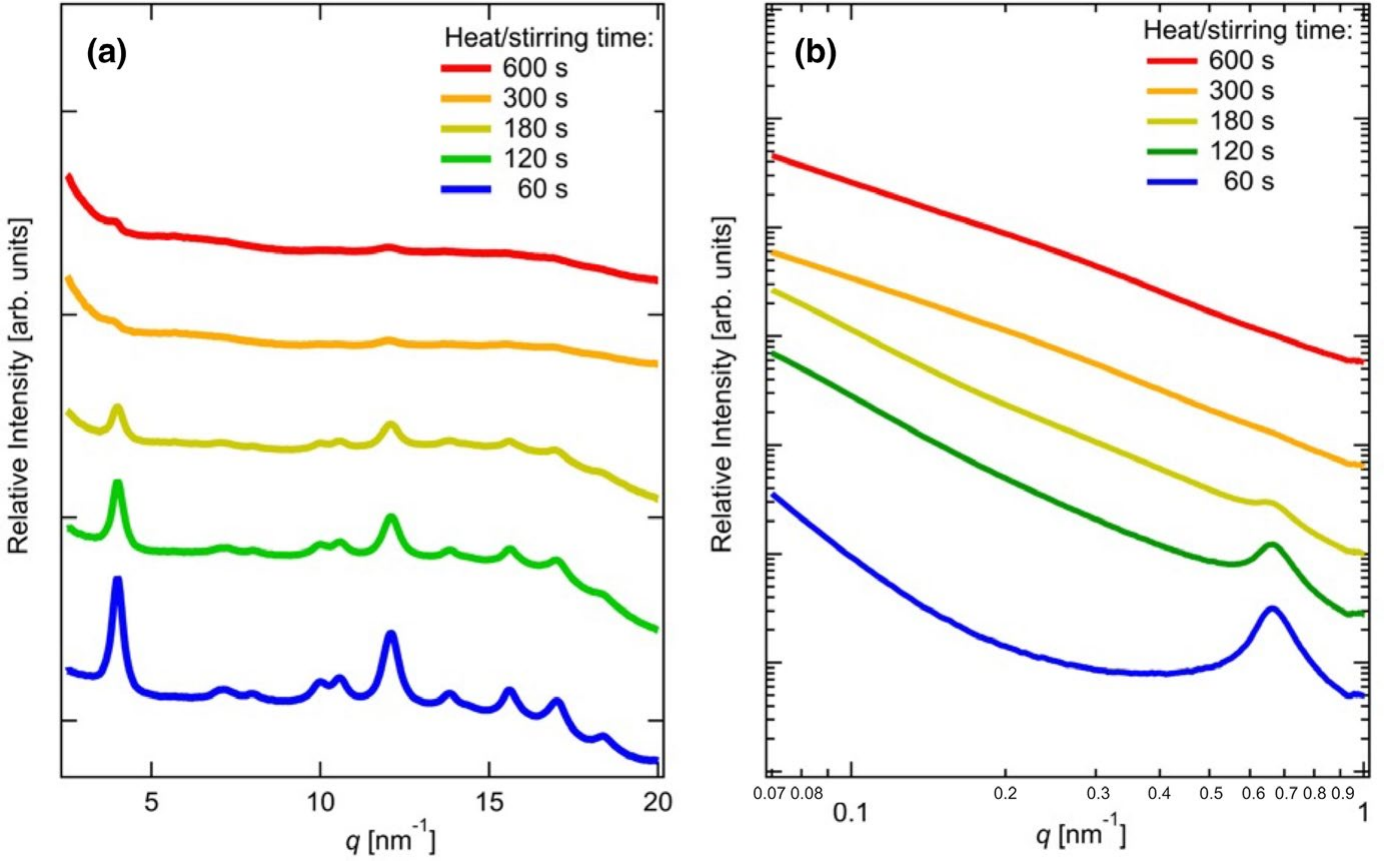
Dependence of heating and stirring times evaluated by analyzing the (a) WAXS profiles and (b) SAXS profiles for the following states: 60 s (suspension), 180 s (nonuniformed aggregates), 240 s (opaque gel), and 420 and 660 s (transparent gel)

We analyzed the structural features of varying concentrations of starch suspensions, aggregates, and gels. Initially, we focused on the evolution of the WAXS and SAXS profiles with the heating and stirring times because they can be measured for crystals and higher‐order structures. Figure 8a,b shows the WAXS and SAXS profiles of starch at a concentration of 0.167 g/cm^3^ at 25°C. When the opaque and transparent glue was cooled, we obtained the opaque and transparent gel (postgelatinization).

Diffraction peaks corresponding to the B‐type crystals are observed at 60 s (suspension state) and 180 s (nonuniform aggregates), as shown in Figure 8a. During heating and stirring, all the diffraction peaks become smaller. Even in the opaque and transparent gel states, only two diffraction peaks at *q* = 4.0 and 12.4 nm^‐1^ are observed. In other words, the starch gel has a crystalline structure at room temperature, and the crystallinity decreases with increasing heating and stirring times. These results suggest that the amylopectin crystals are likely in their molten form during heating and stirring. In Figure 8b, the time evolution of the SAXS profiles is shown. Peaks are observed at *q* = 0.67 nm^‐1^ and *L* = 9.38 nm (Equation 2). The peak positions are independent of time, whereas the peak intensities decrease with time. The results obtained by analyzing the SAXS profiles reveal that the interlamellar amorphous regions do not expand during heating and stirring following the absorption of water molecules. On the other hand, the crystallinity evaluated by analyzing Figure 8a decreases with increasing heat/stirring times. Therefore, these results suggest that the width of the crystal region reduces despite the constant thickness of the crystal and amorphous regions during gelatinization.

The scattering intensity decreases with increasing *q*, in accordance with the power‐law formula expressed in Equation (3), as evidenced by the *k*‐values in Table 1. The SAXS profile analysis of starch suspension and gels is mostly based on the fractal concept (Blazek & Gilbert, 2011). The scattering exponent *k* is 3.01 for the suspension state. From Equations (3) and (4), the surface fraction dimension, *d*_s_, is obtained as 2.99. Owing to the absorption of water molecules and heating/stirring, the surface becomes rough. The surface of the wet granules appears smoother before heating. The constant *k* is in the range of 1–3 for 1–3‐D structures. In the nonuniform aggregates (180 s) and the opaque gel state (240 s), the *k*‐value becomes approximately 2.4. At this stage, the collapse of starch powder is initiated. After the powder disappears, the gel becomes transparent, and the *k*‐value becomes 1.6 (Ren & Zuo, 2018). Gelation processes have been previously studied using the X‐ray scattering technique (Vermeylen et al., 2006). Researchers analyzed the *k*‐values in the high‐*q* region using the SAXS technique (temperature: >90°C). In our study, we have focused on the scattering profiles of the gel state. The *k*‐value was assumed to depend on the scattering of molecular networks, which might be the diffusion‐limited aggregation cluster. For example, the formation of a 2D diffusion‐limited aggregation cluster presented by Anitas (2020) was attributed to the particles undergoing a random walk (attributable to Brownian motion), eventually clustering into aggregates. The *k*‐value obtained by us agrees well with the analytical value obtained by Muthukumar (1983) using the mean‐field theory for diffusion‐limited cluster formation. Furthermore, Anita studied the scattering curves from mass and surface fractals in a 2D Sierpinski Gasket (SG), a diffusion‐limited cluster (Anitas, 2018). The *k*‐value calculated in this case is 1.59, which is comparable to the value obtained by us for a transparent gel.

**TABLE 1:** Dependence of the heating and stirring times on the scattering exponent *k*

Heating/Stirring time (s)	*k*	State
60	3.01	Suspension
180	2.53	Non‐uniformed aggregates
240	2.33	Opaque gel
420	1.59	Transparent gel
660	1.59	Transparent gel

Highly branched amylopectin units are typically the major components in starch granules with α(1–4)‐linked glucose linear chains and α(1–6)‐linked branch points. Amylopectin molecules have many branches (Marchel et al., 2001). In the transparent gel state, the branches can potentially expand. This can be attributed to the melting crystal structure and absorption of water molecules. However, it was difficult to evaluate the diffusion‐limited cluster size using the SAXS technique as the *q*‐window was small. Therefore, USAXS measurements were performed, which were evaluated on a scale between micrometer and nanometer for the transparent gel state under two different conditions.

### 3.4 USAXS measurements for the transparent gel state of potato starch

**FIGURE 9:**
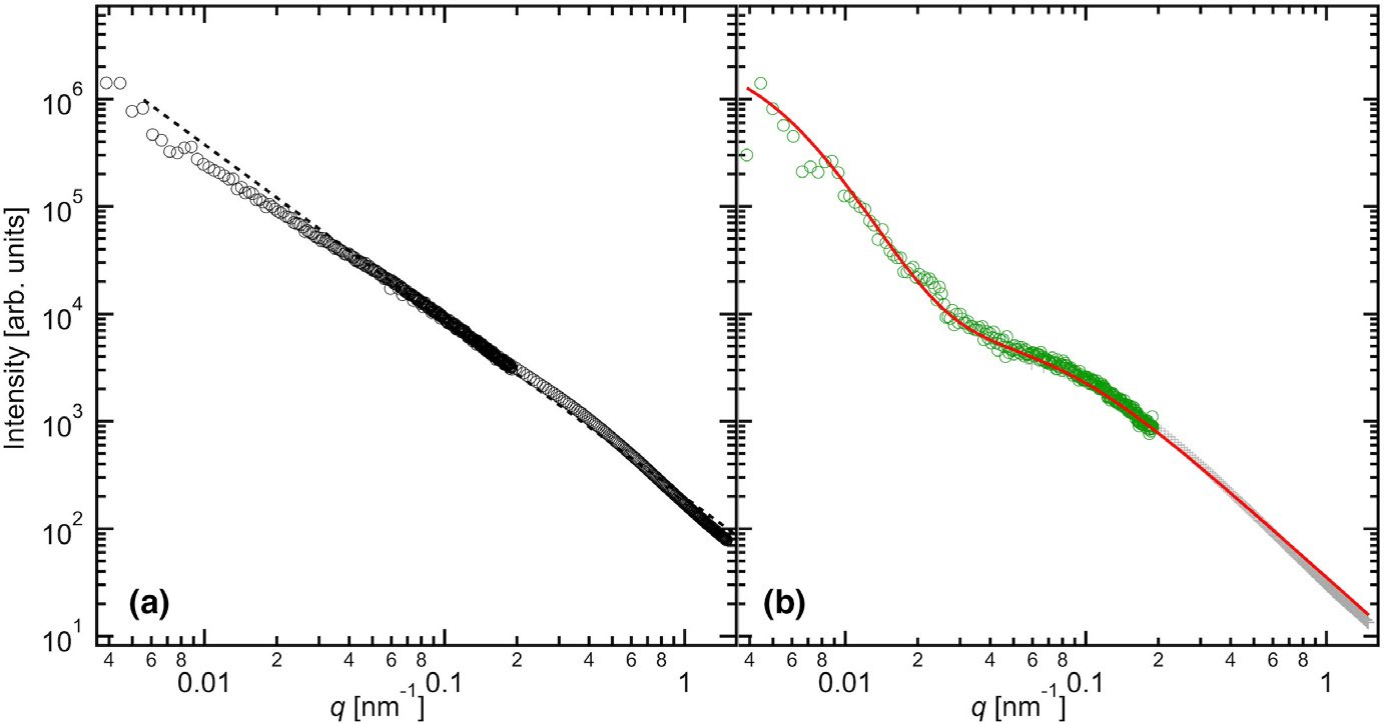
USAXS profiles of the transparent starch gel and the corresponding fitting curves (a) at 0.167 g/cm^3^ (USAXS measurements (open circles) and the *I* ~ *q* ^−1.59^ curve (dashed line)) and (b) at 0.024 g/cm^3^ (USAXS measurements (open circles) and a correlation length fit model (solid line))

Figure 9a,b shows the USAXS profiles of the transparent gel states at 0.167 and 0.024 g/cm^3^, respectively. The comparison of the concentrations of starch and network structure was made under conditions of low concentration. When the value was 0.167 g/cm^3^ (Figure 9a), *k* was 1.59 in the *q* range of 0.06–1.0 nm^‐1^. Below *q* = 0.06 nm^‐1^, the radius of gyration, *R*_g_, was evaluated from the Guinier equation as follows:

(5) $$I\left(q\right)=I\left(q=0\right)\exp\left(‐\frac{1}{3}R_{g}^{2}q^{2}\right).$$

Here, the forward scattering, *I*(*q* = 0), measures the total mass of the object. *R*_g_ is a size parameter and may have a simple relationship with the geometric size. Analysis of the fit curve (Figure S2) reveals that *R*_g_ = 64.3 nm. In this case, the particle might be the 2D diffusion‐limited cluster. Therefore, the *R*_g_ of a disk is assumed to be correlated with its radius (*R*) through $R_{g}^{2}=\frac{R^{2}}{2}$, where *R* = 90.2 nm.

In the case of 0.024 g/cm^3^ (Figure 9b), the characteristic length ξ in microgel networks is evaluated. It describes the decay of liquid‐like correlations in a polymer network (Witte et al., 2019). While ξ is smaller than the mean distance between the chemical crosslinks, it is proportional to the distance between the chemical crosslinks. It is described by the Ornstein–Zernike equation as follows:

(6) $$I\left(q\right)=\left(\frac{A}{1+\xi^{2}q^{2}}+\frac{B}{{\left(1+\Xi^{2}q^{2}\right)}^{2}}\right).$$

The additional terms involving *A* and *B* represent the inhomogeneity of the polymer chain components topologically frozen by cross‐linking. The two correlation lengths, ξ and Ξ, were evaluated as 13 and 180 nm, respectively. The large Ξ value is similar to that reported for the 9‐nm repeat structure and is double the *R* (diameter of the 2D diffusion‐limited cluster; obtained by analyzing the fitting curve of the transparent gel at 0.167 g/cm^3^). This result suggests that an amylopectin molecule exhibits 100‐nm‐scaled fluctuations. A smaller correlation length, ξ, was observed under conditions of low starch concentration (0.024 g/cm^3^) because under conditions of high starch concentration (0.167 g/cm^3^), the 9‐nm repeat structure would be not observed due to scattering derived from the cross‐linked points (as the network structure grows due to gelatinization).

### 3.5 Models of opaque and transparent gels of potato starch

**FIGURE 10:**
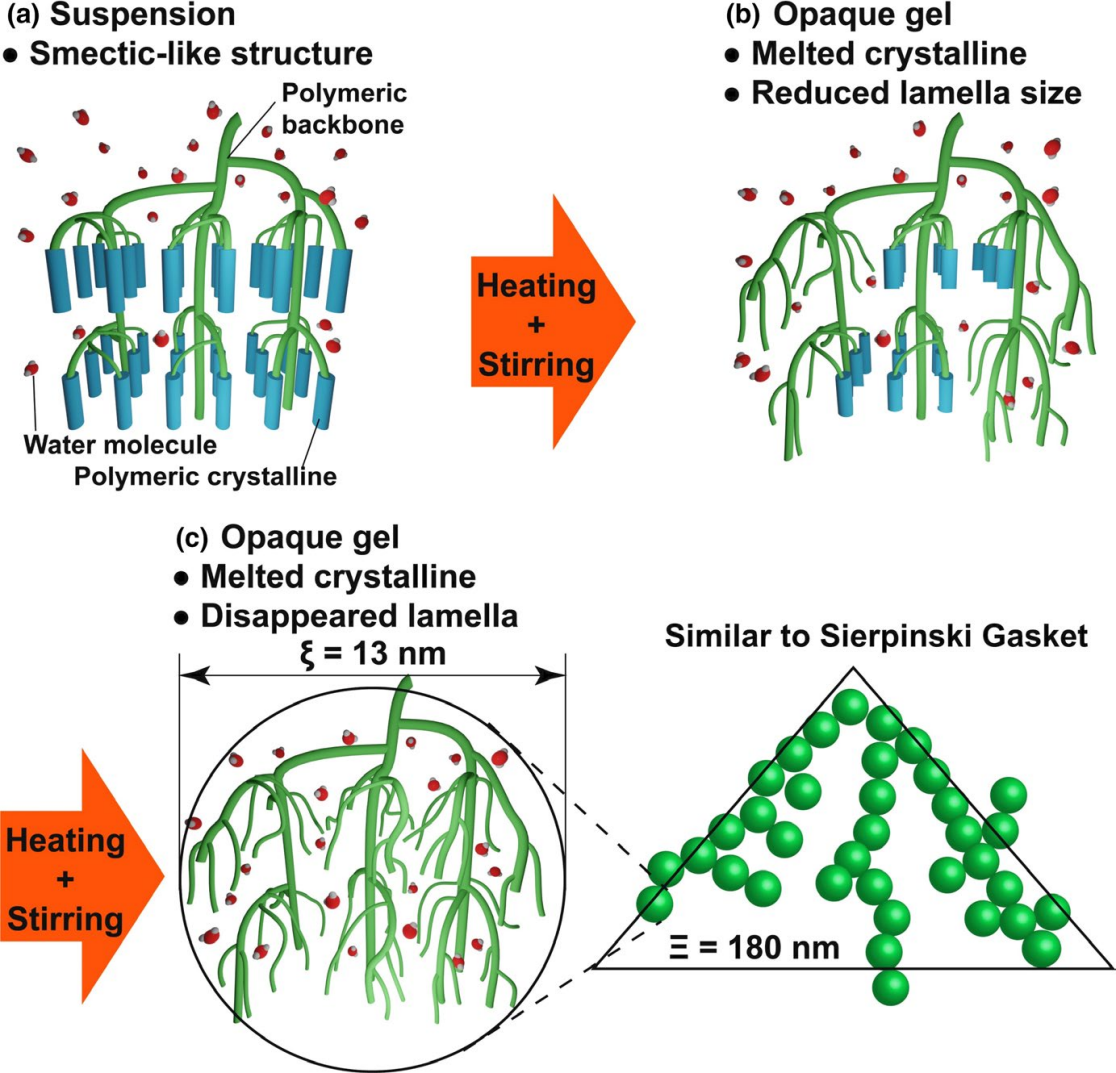
Schematic representation of the transparent gel structure. In nonuniform aggregates and opaque gel state, the 9‐nm repeat structure survives even after the processes of heating and stirring have been conducted. The transparent gel structure has two different correlation lengths, ξ (=13 nm) and Ξ (=180 nm)

Figure 10 shows the model of the opaque and transparent gels of potato starch, starting from a smectic‐like structure. The lamellar crystals in the smectic‐like structure are in their molten forms (Figure 10a), and the size of the lamellar width decreases as the peak intensity from the 9‐nm repeat structure decreases with time. However, it is independent of the correlation length of the lamellar crystals. When the sample is further heated and stirred, the powder structure collapses and changes to that of a small crystallite. At the macroscale, however, the starch powder suspension transforms to an opaque glue state. The opaque gels and the collapse of the powder structure are observed using the SAXS technique when the samples are quenched and the temperature is brought down to approximately 25°C (Figure 10b). These results suggest that the inhomogeneity in the micrometer to nanometer scale is retained in the opaque glue or gel states. A weak peak is observed from a small 9‐nm repeat structure consisting of a very small crystallite. The 9‐nm repeat structure and crystal lattice that are in their molten forms take the transparent glue form upon further heating and stirring. Therefore, amylopectin chains expand and spread molecular chains throughout the system, leading to an increase in viscosity. After quenching to room temperature, the transparent glue state is observed where 9‐nm repeat structures are absent and only small crystals of cross‐linking points of the transparent gel are present. Two different correlation lengths of ξ and Ξ are evaluated as 13 and 180 nm, respectively, under conditions of different concentrations by analyzing the results obtained using the USAXS technique (left of Figure 10c). In particular, the size of the 100‐nm‐scale fluctuations is ~180 nm. Therefore, the amylopectin network is suspected of having a correlation length of 180 nm (the green triangle toward the right of Figure 10c). This length is similar to that observed in a 2D SG (Anitas, 2018). These correlation lengths can potentially be assigned to the 2D diffusion‐limited clusters. The degree of polymerization of glucose (in potato amylopectin) was estimated by Suzuki et al. (1994). The maximum degree of polymerization was >1,000. The entire amylopectin molecule contains ~5–7 units of these 180‐nm‐scale diffusion‐limited clusters. These clusters have small components of ~13 nm, which are similar to those present in the 9‐nm repeat structure. This is the so‐called cluster structure of amylopectin. After the melting of the smectic‐like structure, the network structure expands by absorbing water molecules.

## 4 CONCLUSION

Edible potato starch was studied to understand the interaction present between the granular structure and water molecules. The study was also conducted to understand the changes in the gelation of amylopectin over a wide spatial scale. The dependence of gelation on the potato starch concentration was studied. The gelation rate decreased as the concentration decreased. To evaluate the interaction of amylopectin with water, the humidity dependence of the starch grains was evaluated using the humidity‐controlled FT‐IR spectroscopy technique. The adsorption of water resulted in an increase in the intensities and a shift to a lower wavenumber region for the adsorption of amylopectin molecules. Furthermore, the results obtained using the SAXS technique (during the study of the suspensions) revealed that the phase of the 9‐nm repeat structure changed from nematic‐like to smectic‐like. This can be attributed to the presence of large amounts of water molecules in the amorphous region of the interlamellar amylopectin crystals. Further heating of the starch granules resulted in the gradual collapse and change of nonuniform aggregates and the opaque gel. Further heating and stirring resulted in the formation of a transparent gel. Analysis of the SAXS profiles revealed that immediately after heating, a highly disordered surface structure was formed for the suspension. The grains started to break up from the nonuniform aggregates. In this case, the 9‐nm repeat structure was observed on the nonuniform aggregates and the opaque gel, which indicated the correlation of the lamellar crystals even in the opaque gel state. The correlation length did not change with heating. In other words, only the size of the lamellar crystal width decreased. In the case of the transparent gel, the scattering exponent was 1.59 and the structure was a 2D diffusion‐like cluster. The structure was precisely determined using the USAXS technique. The 13‐nm‐size clusters were observed to form a network of a 2D diffusion‐like cluster structure of size 180 nm. Thus, the gelation of starch was efficiently studied.

## CONFLICT OF INTEREST

The authors have no conflicts of interest directly relevant to the content of this article.

## Supporting information

Supplementary Material

## Data Availability

The data that support the findings of this study are available from the corresponding author, Go Matsuba, upon reasonable request.
