# Supplementary material for: Analysis of the sol and gel structures of potato starch over a wide spatial scale
Source: Food Sci Nutr. 2021 Jul 21;9(9):4916–26. doi: 10.1002/fsn3.2441 (PMC8441370; doi:10.1002/fsn3.2441)
Supplement: Supplementary file 1 — Supplementary Material [file FSN3-9-4916-s001.docx]

**Supporting Information**

Analysis of sol and gel structures of potato starch over a wide spatial scale

Akane NAGASAKI^1^, Go MATSUBA^1,*^, Yuka IKEMOTO^2^, Taro MORIWAKI^2^, Noboru OHTA^2^, Keiichi OSAKA^2^

^1^Graduate School of Organic Materials Engineering, Yamagata University, 992-8510 Japan

^2^SPring-8/JASRI, 679-5198 Japan

**1. Microbeam FT-IR measurements.**

The detailed information about microbeam FT-IR measurements is described in ref. [14]. Figure S1(a) shows the photo of microbeam FT-IR spectrometer with the humidity control cell. The image of granular starch sample is shown in Figure S1(b). The scale bar is 5 μm.

**Figure S1.** (a) The photos of microbeam FT-IR spectrometer in BL43IR, SPring-8. (b) The photo of the starch granule sample. The scale bar is 5 μm.

2. Guinier plot of the transparent gel of potato starch

We evaluated the radius of gyration from USAXS profiles. Figure S2 shows the Guinier plot (log *I*(*q*) vs. *q*^2^) from *q*^2^ = 1.22 x 10^-5^ nm^-2^ to 3.0 x 10^-3^ nm^-2^. From these results, we could evaluate *R*_g_ = 64.3 nm for each sample from the equation (5).


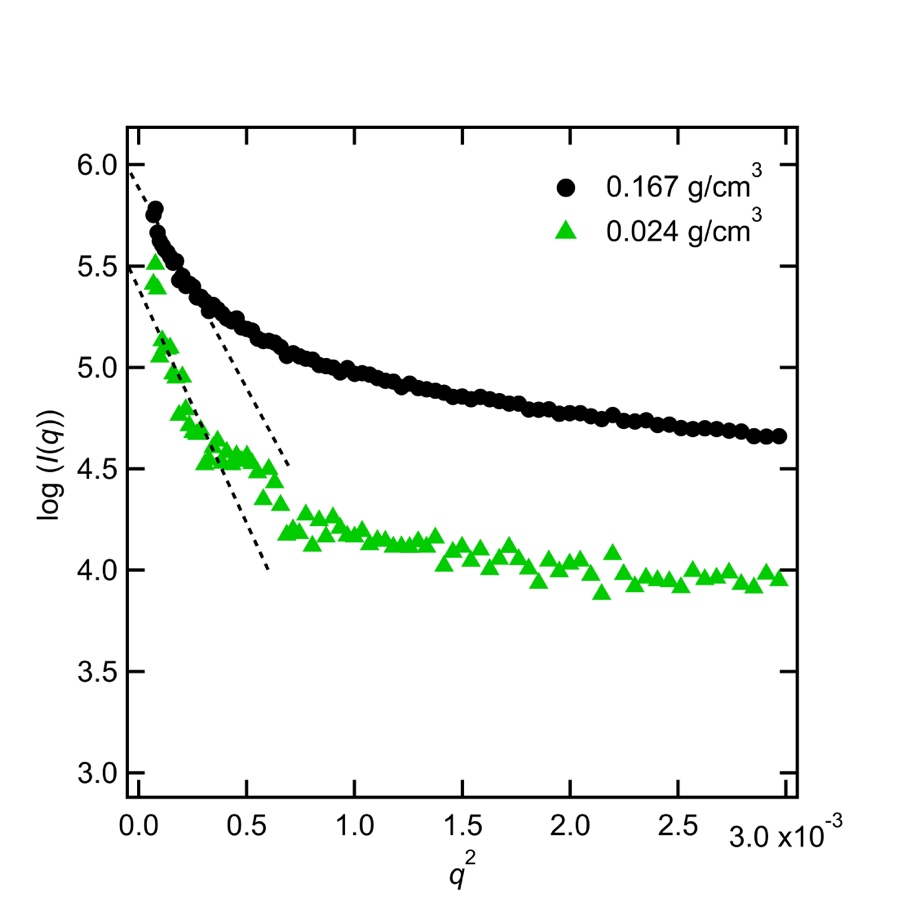


**Figure S2.** Guinier plot of USAXS profiles of the transparent starch gel. The dashed line is corresponding fitting curves at 0.167 g/cm^3^ and 0.024 g/cm^3^.
